# Linear quantification of lymphoid infiltration of the tumor margin: a reproducible method, developed with colorectal cancer tissues, for assessing a highly variable prognostic factor

**DOI:** 10.1186/1746-1596-7-156

**Published:** 2012-11-13

**Authors:** Marc-Antoine Allard, Jean Baptiste Bachet, Alain Beauchet, Catherine Julie, Robert Malafosse, Christophe Penna, Bernard Nordlinger, Jean-François Emile

**Affiliations:** 1 EA4340, Versailles SQY University, Boulogne, France; 2 Department of Digestive and Oncological Surgery, Ambroise Paré Hospital, APHP, Boulogne, France; 3 Department of Digestive Oncology, Ambroise Paré Hospital, APHP, Boulogne, France; 4 Department of Clinical Research, and Ambroise Paré Hospital, APHP, Boulogne, France; 5 Department of Pathology, Ambroise Paré Hospital, APHP, 9 Av. Charles de Gaulle, Boulogne F-92104, France; 6 present address: Hôpital de la Pitié, APHP, Paris, France; 7 present address: Hôpital de Bicêtre, APHP, Bicêtre, France

**Keywords:** Tumor infiltration, Lymphocytes, Invasive margin, Linear quantification, Colorectal cancer, Image analysis, Automated count

## Abstract

**Background:**

Lymphoid infiltration is a prognostic marker in solid tumors, such as colorectal, breast and lung carcinomas. However, lymphoid infiltration is heterogeneous and the reproducibility of quantification based on single counts within a tumor is very low. We aimed to develop a reproducible method for evaluating lymphoid infiltration in tumors.

**Methods:**

Virtual slides were obtained from tissue sections from the localized colorectal carcinomas of 117 patients, stained for CD3 and CD45R0. We assessed the variation of lymphoid cell density by automatic counts in 1 mm-wide, 5 μm-long segments of the invasive front, along an axis 4 mm in length running perpendicular to the invasive front of the tumor.

**Results:**

We plotted curves of the variation of lymphocyte density across the tumor front. Three distinct patterns emerged from this linear quantification of lymphocyte (LQLI). In pattern 1, there was a high density of lymphocytes within the tumor. In pattern 2, lymphocyte density peaked close to the invasive margin. In pattern 3, lymphocytes were diffusely distributed, at low density. It was possible to classify all the tumors studied, and interobserver reproducibility was excellent (kappa =0.9). By contrast, single counts of CD3^+^ cells on tissue microarrays were highly variable for a given LQLI pattern, confirming the heterogeneity of lymphoid infiltration within individual tumors. In univariate analysis, all pathologic features (stage, metastatic lymph node ratio (LNR), vascular embolism, perineural invasion), CD3^+^ cell density, LQLI patterns for CD3^+^ and CD45R0^+^ cells) were found to have a significant effect on disease-free survival (DFS). In multivariate analysis, only the LQLI pattern for CD3^+^ cells (HR: 6.02; 95% CI: 2.74-13.18) and metastatic lymph node ratio (HR: 6.14; 95% CI: 2.32-16.2) were associated with DFS.

**Conclusion:**

LQLI is an automated, reproducible method for the assessment of lymphoid infiltration. However, validation of its prognostic value in larger series is required before its introduction into routine practice for prognostic evaluation in patients with colorectal carcinomas.

**Virtual slides:**

The virtual slide(s) for this article can be found here: http://www.diagnosticpathology.diagnomx.eu/vs/9861460717895880

## Background

It is now clearly established that tumor progression depends not only on the biological characteristics of the tumor, but also on the host immune response to the tumor [1,2]. More than four thousand papers have been published on the immune response and carcinomas over the last 40 years. Most studies of this aspect in solid cancers, mostly colorectal carcinoma (CRC), lung and breast cancers, reported a strong correlation between the density of immune cells, particularly lymphocytes, within tumors and long-term survival [3-10]. However, despite this massive body of evidence, lymphocyte infiltration (LI) is not taken into account in daily practice, even for discussions of adjuvant treatment in patients with stage II or III CRC. This paradox is explained by the lack of a reproducible and suitable method for assessing LI. There is, therefore, a need to develop new, more reproducible methods for LI assessment.

Immune cell density is evaluated on slides stained with hematoxylin and eosin (H&E) or by *in situ* immunohistochemistry on tumor samples, performed on whole slides or tissue microarrays (TMA) [11]. In early studies of lymphocyte infiltration, the pathologists counted the lymphocytes within a limited area of the tumor themselves, by eye [3,4]. However, this method was time-consuming and subject to human error, the area of the tumor considered was small and reproducibility between pathologists was poor. The more recent development of a combination of virtual slide technology and image analysis software has made high-throughput counting possible. This approach has been adopted by several authors, mostly using TMA [5,12]. Lymphocyte density is classified as “high “or “low”, depending on whether the absolute number of lymphocytes obtained is higher or lower than the mean absolute number of lymphocytes for the study population.

TMA-based approaches are cheap and pertinent in this field, but the area analyzed is limited to the size of the TMA core (diameter of 0.6 mm in most studies).

Furthermore, LI is highly heterogeneous within tumors, rendering lymphocyte counts in limited areas highly variable. The establishment of a cutoff value for lymphocyte density for the estimation of prognosis is, thus, necessarily biased, and the assessment of tumor lymphoid infiltration based on absolute values for restricted areas is unsatisfactory. The aim of this study was to develop a reproducible method for LI quantification, overcoming these problems of variability.

Recent studies of CRC have shown that the prognostic value of LI determinations can be increased by combining lymphocyte densities within and outside the tumor. This suggests that the environment immediately around the tumor should be included in the evaluation of LI [13].

Based on these findings and the need to limit the bias described above, any method for measuring LI must take large areas of tissue sections, including both tumor tissue and the adjacent region, into account. Such methods should also involve automated counting, based on image analysis, to save time and to overcome potential problems of interobserver variability.

We focused on the CRC CD3^+^ and CD45R0^+^ lymphocyte subpopulations for the development of this method, because infiltration with these subpopulations has been widely reported to have an impact on the outcome of CRC [11,13]. We plotted curves of infiltration along an axis running parallel to the invasive front of the tumor and looked for common patterns in a sample of CRC. We then assessed the prognostic value of this method by comparing the results obtained with those for the prognostic markers used in routine practice and with single counts of CD3^+^ cells on TMA.

## Methods

The first step in this study was the establishment of the linear quantification of lymphocyte infiltration (LQLI) method.

### Samples

**Figure 1:**
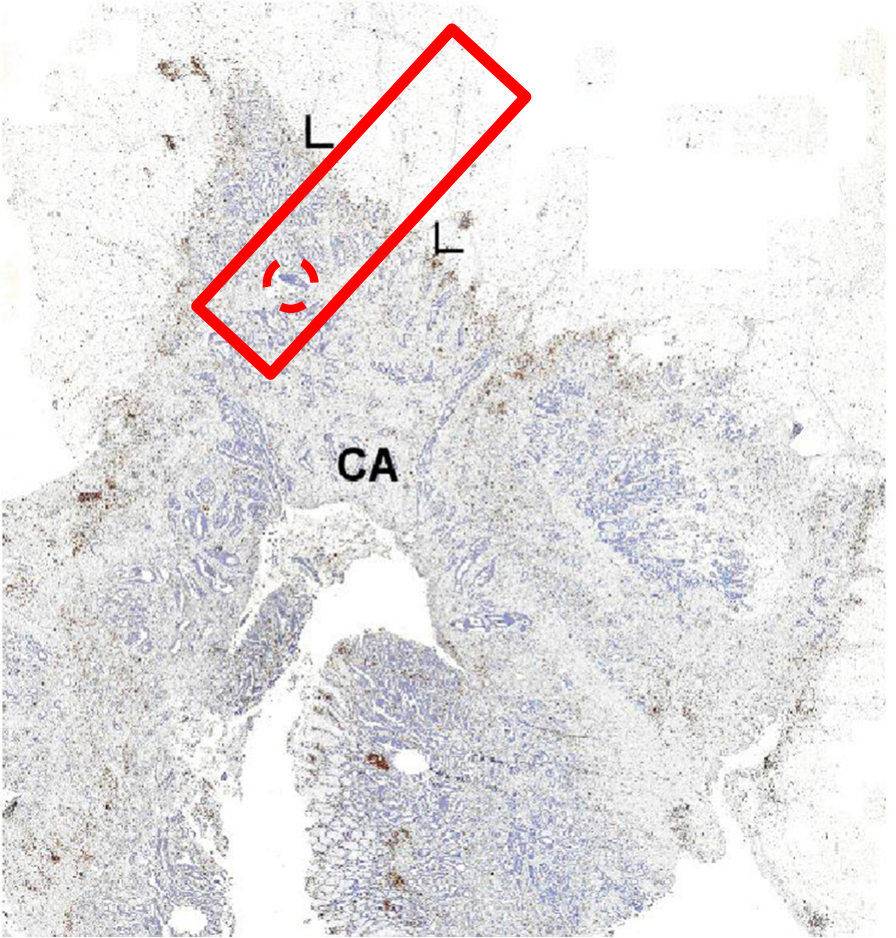
***In situ *immunohistochemistry with brown staining of CD3 lymphocytes and blue nuclear staining of all cells including the invasive front (arrows) of a colon adenocarcinoma (CA).** The rectangle represents surface of analysis of our method and dotted circle correspond to TMA core (0.6mm).

H&E-stained slides of CRC sections were checked for the presence of an invasive tumor front. Formalin-fixed paraffin-embedded (FFPE) blocks containing tumor tissue with an invasive front and surrounded tissue were selected for the construction of TMA and the application of our quantification method (see below; Figure 1). Paraffin-embedded samples were obtained from the Pathology Department and the tissue bank of Ambroise Paré Hospital, which has been registered with the French Ministry of Research (# DC 2009–933).

### Immunohistochemistry

Immunohistochemistry was performed on 4 μm-thick section of FFPE tissue samples, with a Bond autostainer (Leica, Biosystems Newcastle Ltd), after antigen retrieval in citrate buffer pH6 (Leica, Biosystems Newcastle Ltd) and endogenous peroxidase inhibition with 3% H_2_O_2_. The primary antibodies were mouse monoclonal anti-CD3 (1/50, clone F7.2.38, Dako France S.A.S.) and anti-CD45R0 (1/50; clone UCHL1, Dako France) antibodies. Antibody binding was detected and the signal was amplified with the Bond Polymer Refine Detection system (Leica, Biosystems Newcastle Ltd) and hematoxylin counterstaining.

### Linear quantification of lymphoid infiltration of the tumor front

#### Support

Virtual slides were obtained by scanning each slide with Mirax Desk (Zeiss, Germany) and quality control with Miraxviewer software (Zeiss, Germany). Images were analyzed with Visilog 9.0 software (Noesis, Saclay, France), a commercially available image analysis suite already used in a previous study [14].

#### Concept

Visilog 9.0 software was specifically developed to express lymphocyte density as a function of the distance from the invasive front of the tumor, defined as the microscopic interface between the normal tissue of the host and the tumor mass. In budding tumors, the tumor front was localized close to areas containing groups of at least five cohesive tumor cells. Lymphocyte density was evaluated from the outside to the inside of the tumor, along an axis perpendicular to the tangent of the invasive front of the tumor.

**Figure 2:**
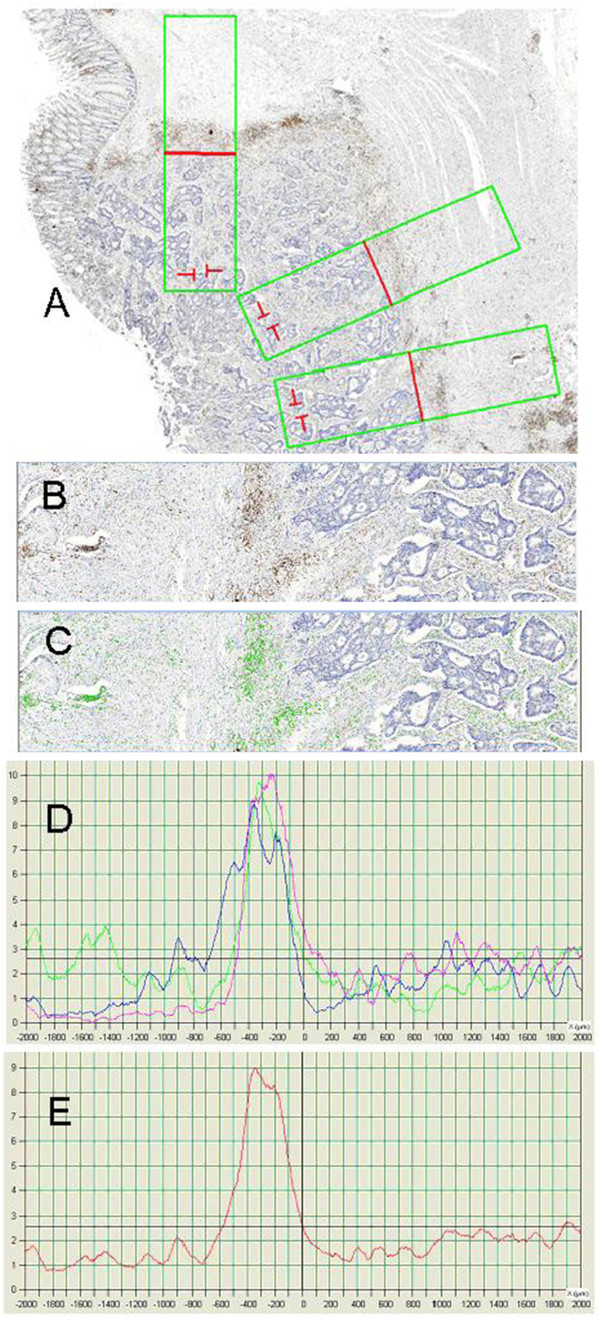
**Linear quantification of lymphocytes (LQL) was performed on virtual slides (A) on one to eight rectangles of 1x4 mm placed across the tumor invasive front.** Each selected area was perpendicular to the invasive front, and the 2 mm of tumor were always to the right (**B**). CD3 positive cells (green stain) were identified by image analysis (**C**). Control was performed by comparing both images (**B** and **C**). Automated quantification of lymphocyte density was then performed every 5 μm from 2 mm outside to 2 mm inside the tumor, on 2 to 8 selected areas of the tumor. Each value corresponded to a surface of 500 μm2, and was associated with the distance from the tumor invasive front, corresponding to the x and y axis of the curve respectively. Each of the different colored curves (blue, green, red) is issued from one area of measurement (**D**). These different curves lead to a mean curve, which was used for the identification of the LQL pattern (**E**). The number of area of measurement varies from two to eight depending on the size of the tumor invasive front.

The area analyzed was a rectangle (1 mm × 4 mm), measuring 4 square millimeters (Figure 1). The invasive front of the tumor was manually positioned, by the observer, in the middle of the rectangle, with the tumor to the right. Lymphocyte cell density was determined for segments of 5 μm. CD3^+^ cells were counted as a function of their distance from the invasive front of the tumor, in 5 μm segments from the outside to the inside of the tumor (Figure 2). We named this method the linear quantification of lymphocyte infiltration (LQLI). As counts were standardized for an area 1 mm wide, each lymphocyte density value corresponded to an area of 5000 μm^2^, and was associated with a distance from the invasive front of the tumor. We then plotted curves of lymphocyte density from the outside to the inside of the tumors, across the invasive front (Figure 2). We ensured that these curves were comparable between patients by positioning the curve on the axes such that the region surrounding the tumor extended from 0 to 2 mm and the tumor region extended from 2 mm to 4 mm.

#### Process

In practice, the process was performed as follows:

1.  Opening the file for the virtual slide in .mrx or .vms format

2.  Automatically generating a 1 × 4 mm rectangle, with preformatted counting areas, measuring 1 mm × 5 μm, along an axis running perpendicular to the tumor front

3.  Orienting the rectangle and positioning it manually on the virtual slide

4.  The simultaneous use of several rectangles on the same virtual slide was possible

5.  Automatic detection of lymphocytes based on a three-step process: i) detection based on color thresholding; ii) boundary definition with a watershed approach and iii) filtering on the detected elements on the basis of size

6.  Visual validation of the identified lymphocytes. It is possible to modify the default parameters beforehand (size, blue intensity, red intensity), if necessary, and to check corresponding results as many times as necessary. The three parameters listed here were identified as the most critical during the evaluation of robustness.

7.  Up to 100 slides can be prepared before automatic counting.

8.  Automatic counting of the lymphocytes in 5 μm segments at known distances from the tumor front. Processing time of 40 to 200 seconds per slide, depending on the number of rectangles and the computer used.

9.  Automatic generation of a curve of distance from the tumor front (*x*) versus lymphocyte count (*y*). The curves for the various rectangles can be merged and used to generate a curve of mean counts.

Manual export of the data in .csv format is possible, for further analyses.

We then compared the prognostic value of LQLI with those of widely used clinical/pathologic parameters, and with CD3^+^ lymphocyte densities determined from TMA cores.

### CD3^+^ lymphocyte densities measured with tissue microarrays

TMA blocks were constructed as follows: three cores, each with a diameter of 0.6 mm, were taken from the tumor region identified on H&E-stained slides (Figure 1) generated from another FFPE block of the same tumor. A manual tissue-array instrument (Beecher Instruments, Alphelys, Plaisir, France) was used to insert each core into the recipient paraffin block. All positive cells within each core were counted automatically and a mean value for the three cores was obtained in square millimeters and used for statistical analysis.

### Evaluation cohort

We studied 117 consecutive patients with stage II and III colorectal cancer (International Union Against Cancer [UICC], TNM classification) undergoing “en bloc” resection between January 1998 and December 2008 at Ambroise Paré Hospital (Table 1). Patients with stage II and III CRC constitute a heterogeneous group with a risk of relapse of 15 to 60%, for which improvements in the assessment of prognosis would be of clinical benefit, particularly as concerns decisions relating to adjuvant therapy. None of the patients with stage II CRC received adjuvant treatment. All patients with stage III CRC completed six months of a FOLFOX adjuvant therapy regimen, in accordance with international recommendations [15,16].

**Table 1:** Description of the study population

**Clinical & pathological characteristics**	** No.**
Mean (s.d.) Age (years)	66 (±12)
Sex	
Male	68 (58)
Female	49 (42)
Mean (s.d.) follow-up (months)	57 (±25)
Median follow-up (range)	61 (5-113)
Tumor stage	
T1	3 (2)
T2	9 (8)
T3	82 (69)
T4	13 (11)
Node stage	
N0	78 (67)
N1	39 (33)
Mean (s.d.) number of LN harvested	27,5 (±14)
Microsatellite instability status	
Positive	10 (9)
Negative	107 (91)
Number of CD3^+^ lymphocytes/mm² (TMA)	
Mean	612 (±481)
Range	21-3014

*LN* lymph node.

The values shown in brackets are percentages, unless otherwise indicated.

The cohort was rendered more homogeneous cohort by the exclusion of patients undergoing palliative resection, or with tumor perforation, a personal history of familial adenomatous polyposis coli, previous chemotherapy and radiotherapy or a tumor of the low or mid rectum.

Follow-up consisted of a physical examination, carcinoembryonic antigen (CEA) determination, CT scan or the thorax and abdomen and/or abdominal ultrasound and chest X ray every three months for the first three years and every six months for two years, as recommended in the Guidelines of the French Society for Gastroenterology, 2008).

This study was approved by the relevant ethics committee, CPP Ile de France 8 (DC 11 05 45).

### Pathology data

All patients included were reviewed for pathological features by two pathologist expert in digestive oncology (CJ & JFE). Immunostaining for mismatch repair proteins was carried out to diagnose microsatellite instability (MSI) as previously reported [17]. Table 1 summarizes clinical and pathological characteristics of the cohort.

### Statistical analysis

Interobservation reproducibility was assessed by calculating the kappa coefficient. The means of three populations were compared by non parametric Kruskal-Wallis tests.

Disease-free survival (DFS) was used as the end-point for prognostic assessment. DFS has been shown to be an appropriate surrogate end-point for overall survival in studies of nonmetastatic colorectal cancer [18]. In survival analysis, we also assessed widely used prognostic factors such as TNM stage, metastatic lymph node ratio (LNR) [19], MSI status, vascular embolism and perineural invasion. We also included the CD3^+^ cell density measured on TMA (Patients were classified as “High LI density” or “Low LI density” with respect to the mean lymphoid density measured with TMA), and LQLI pattern with immunostaining for CD3 and CD45R0. The failure event for DFS analysis was defined as death or relapse. Survival time was calculated from the date of surgery to the date of the failure event or last follow-up. The significance of the effect of the variables tested on DFS was assessed by univariate analysis, with log-rank tests. We used a Cox proportional hazards model to assess the prognostic value of all variables identified as significant in univariate analysis. A *P* value ≤ 0.1 was required for entry into the model. Statistical significance was indicated by a *P* value < 0.05. The analysis was conducted with R software (2.14.1), except for Kaplan-Meier curves, which were plotted with IBM SPSS Statistics 20.

## Results

### LQLI patterns for CD3^+^cells

It was possible to apply the LQLI method to all tumors. The LQLI curves for each tumor were all very similar, suggesting that tumors had a specific pattern of lymphoid cell density variation across the invasive front.

**Figure 3:**
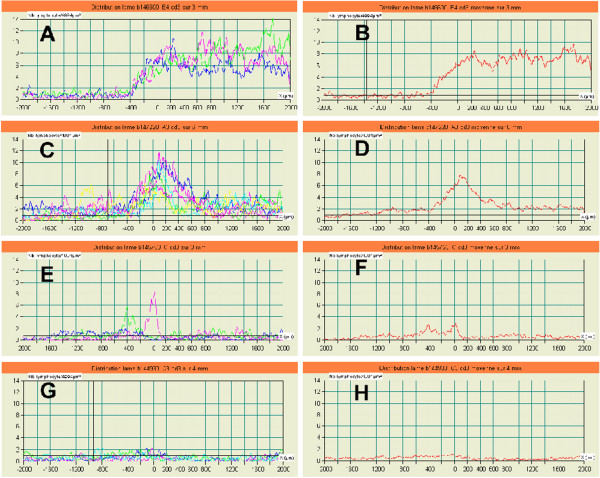
**Left side (A, C, E, G): Curves derived from different measures performed on 4 tumors.** Right side (**B, D, F, H**): Different pattern of lymphocytic infiltration defined according to the mean curve derived from the different measures in A, C, E and G respectively. Pattern 1: High density of positive lymphocytes within the tumors (**A, B**). Pattern 2: High Peak of lymphocyte density within the non-tumorous tissues localized nearby the tumors (**C, D**) and Low Peak of lymphocyte density within the non-tumorous tissues localized nearby the tumors (**E, F**). Pattern 3: Uniform low lymphocyte density (**G**, **H**).

We then tried to define groups of tumors with similar LQLI. Comparisons of the curves obtained led to the identification of three distinct patterns. In the first (pattern 1), lymphocyte density was higher within the tumor than in the region surrounding the tumor. This pattern accounted for 21% of the study population (Figure 3; A, B).

The second pattern was marked by the existence of a significant peak in the invasive front region. This feature corresponded to the presence of large numbers of lymphocytes in the close vicinity of the tumor. Lymphocyte density increased just ahead of the invasive front, at a mean distance of 400 μm ahead of this front, and declined in the 200 μm beyond the invasive front. This pattern was observed in 61% of patients. The height of the peak was varied considerably between patients, from 3 to 14 CD3^+^ cells/5000 μm^2^ (Figure 3; C, D, E, F) with a mean value of 6.7 CD3^+^ cells/5000 μm^2^.

In the third pattern, the density of CD3^+^ lymphocytes was low throughout the tumor front, with no significant fluctuation. This pattern was detected in 18% of the patients (Figure 3; G, H).

### LQLI pattern for CD45R0^+^ cells

The same three LQLI patterns were observed with CD45R0 immunostaining.

In 104 patients (89%), the type of LQLI pattern was identical with both antibodies. Discrepancies between the LQLI pattern for a given tumor were observed in 13 patients (11%) and mostly concerned patterns 2 and 3 (Table 2).

**Table 2:** **Discrepancies between the LQLI patterns for CD3**^**+ **^**and CD45R0**^**+ **^**cells**

**Number of patients**	**CD45R0**^**+**^	** CD3**^**+**^
4	Pattern 2	Pattern 3
1	Pattern 1	Pattern 2
6	Pattern 3	Pattern 2
2	Pattern 2	Pattern 1

### Heterogeneity of tumor lymphoid infiltration

**Figure 4:**
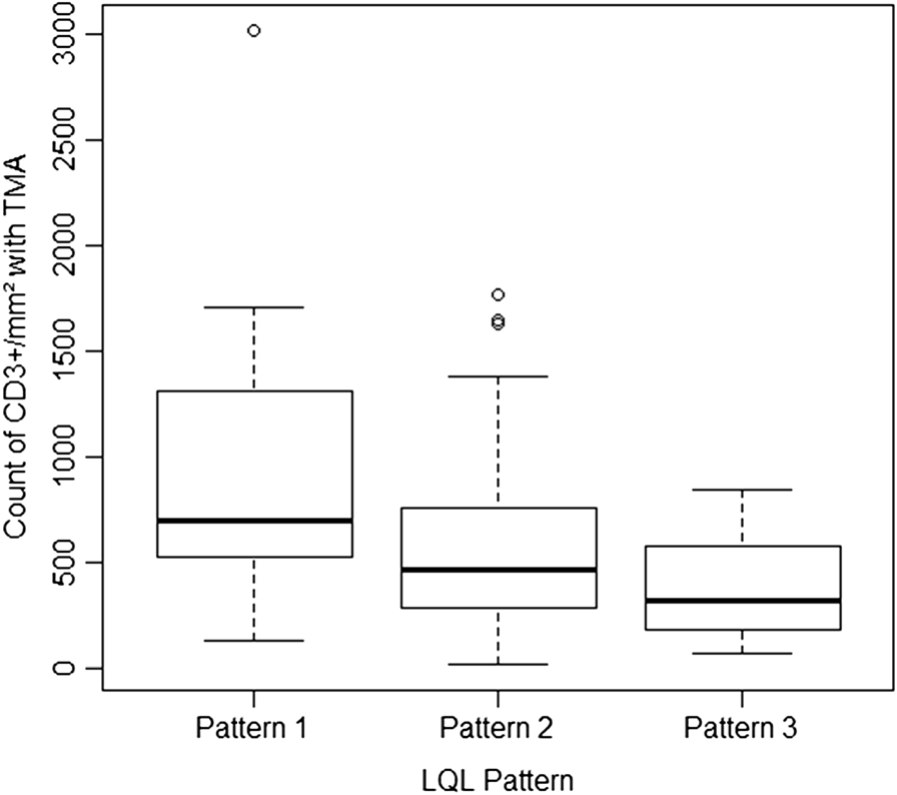
Distribution of CD3+ density according to the LQL pattern.

Mean lymphocyte density, as assessed by automatic counting on TMA, was 612 CD3^+^ cells/mm^2^ (range, 21 to 3014). Mean CD3^+^ cell densities were 922/mm^2^ (range, 132 to 3014), 581/mm^2^ (21 to 1768) and 392/mm^2^ (67 to 846) for patients displaying LQLI patterns 1, 2 and 3, respectively. The differences between the mean CD3^+^ cell densities for these three patterns were significant (*P* = 0.002; Figure 4).

**Figure 5:**
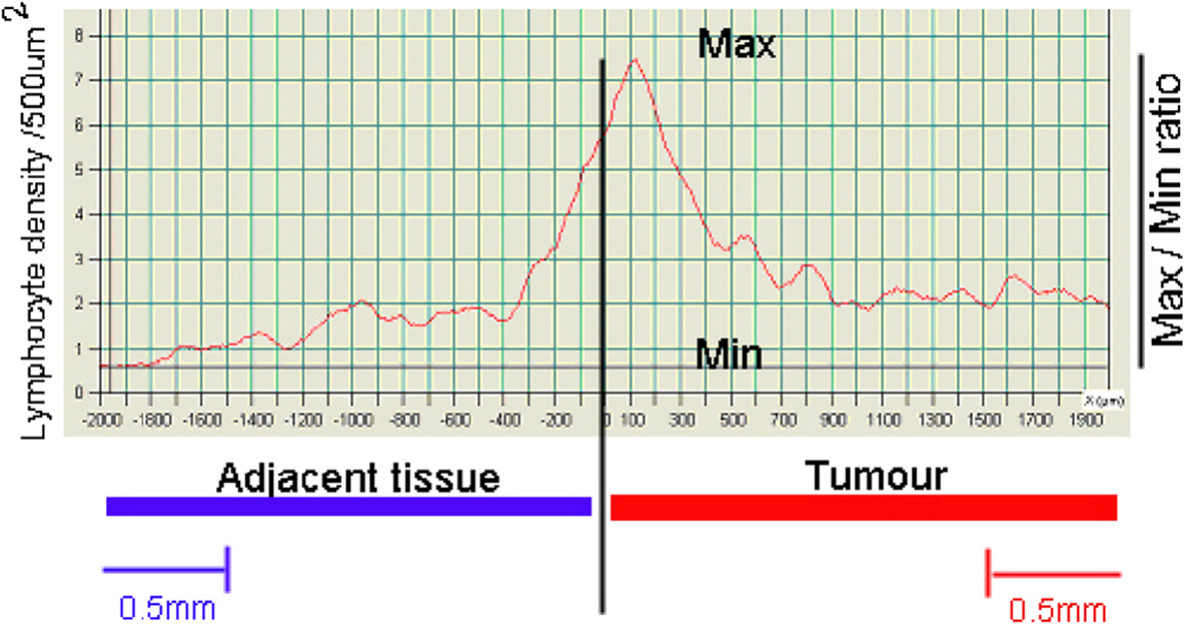
**The ratio of the maximal and minimal CD3+ cells density was evaluated for all tumors.** The variation was significantly lower when evaluated more than 1.5 mm away from the tumor invasive front.

The fluctuations in the parts of the curve corresponding to the tumor and the surrounding tissue highlighted the considerable variability of The maximal/minimal tumor CD3^+^ cell density ratio within each tumor ranged from 2.5 to 55, with a median value of 14.5. Heterogeneity was lower at greater distances from the invasive tumor front. Indeed the median maximal/minimal CD3^+^ cell density ratios for points 1.5 mm outside and 2 mm within the tumor were 2.2 (1.2 to 3.5) and 2.4 (1.25 to 4), respectively (Figure 5).

### Reproducibility

The robustness of the method was confirmed by analyzing sections from different paraffin-embedded blocks for a given tumor, carrying out CD3 immunostaining separately on several sections of a given block and generating several virtual slides by multiple scans of a small number of slides. We found that LQLI measurements at different points along the length of the tumor front had no effect on the pattern observed (Figure 2).

The reproducibility of pattern classification was assessed by the analysis of 50 randomly chosen CD3^+^ cell density curves by a surgeon with limited experience in pathology (MAA) and by two experienced pathologists (JFE & CJ). Interobserver reproducibility was excellent (kappa = 0.93). The mean time required for LQLI and classification of the LI pattern by a pathologist was less than 2 minutes.

### Prognostic value of LQLI pattern

After a median follow-up of 61 months (range, 5 to 113), 24 patients (20.5%) had died or relapsed, and 93 patients (79.5%) were alive without recurrence.

Univariate analysis showed that pT stage and pN status, the total number of lymph nodes, vascular embolism and perineural invasion were significant prognostic factors. LI, assessed by either CD3 count on TMA or by LQLI, was also a prognostic factor. Ten patients had CRC with high levels of MSI. Four of these patients had LQLI pattern 1, five had LQLI pattern 2 and one had LQLI pattern 3. None of the patients with high-MSI tumors relapsed, but MSI status was not identified as a significant prognostic value in this series of 117 patients. These results and the corresponding three- and five-year DFS results for each variable are reported in Table 3.

**Table 3:** **Univariate analysis of pathological variables and two automated techniques (CD3**^**+ **^**cell counts from TMA and LQLI) for disease-free survival**

**Clinical & pathological variables**	**No.**	**%**	**3-yr DFS**	**5-yr DFS**	**HR**	**95% CI**	***P***
TNM stage					2.97	1.29-6.81	0.009
II	78	67	91	87			
III	39	33	74	67			
Metastatic LNR ratio					4.49	1.81-11.13	<0.001
≤0.2	102	87	89	85			
>0.2	15	13	58	58			
Vascular embolism					4.05	1.75-9.37	0.001
Yes	41	35	73	66			
No	76	65	92	89			
Perineural invasion					2.96	1.28-6.85	0.01
Yes	25	21	67	60			
No	92	79	90	86			
MSI status					1.36	0.89-2.34	0.11
MSI -	107	91	84	80			
MSI +	10	9	No event	No event			
Lymphocyte density (CD3+ cells/mm²) with TMA					2.54	1.1-6.4	0.04
High [CD3+]	50	43	92	90			
Low [CD3+]	67	57	80	74			
CD3+ LQLI pattern					4.75	2.33-9.68	<0.0001
Pattern 1	24	20	96	96			
Pattern 2	71	61	90	86			
Pattern 3	22	19	59	45			
CD45R0+ LQLI pattern							
Pattern 1	23	19	96	96	3.85	1.09-7.77	<0.001
Pattern 2	70	60	90	85			
Pattern 3	24	21	62	44			

*LNR* lymph node ratio, *HR* hazard ratio, *CI* confidence interval, *DFS* disease-free survival.

**Figure 6:**
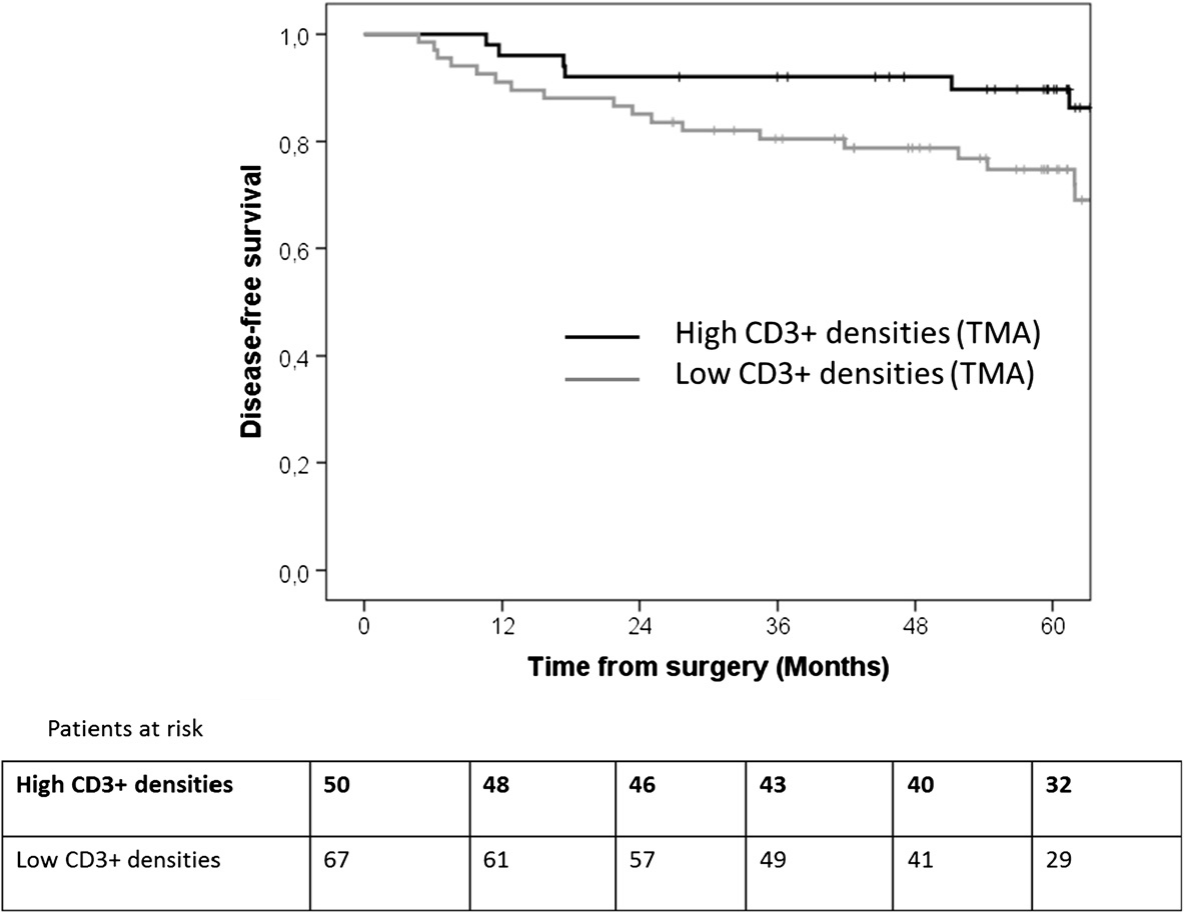
Kaplan Meier disease-free survival curve according to CD3+ densities measured on TMA cores.

**Figure 7:**
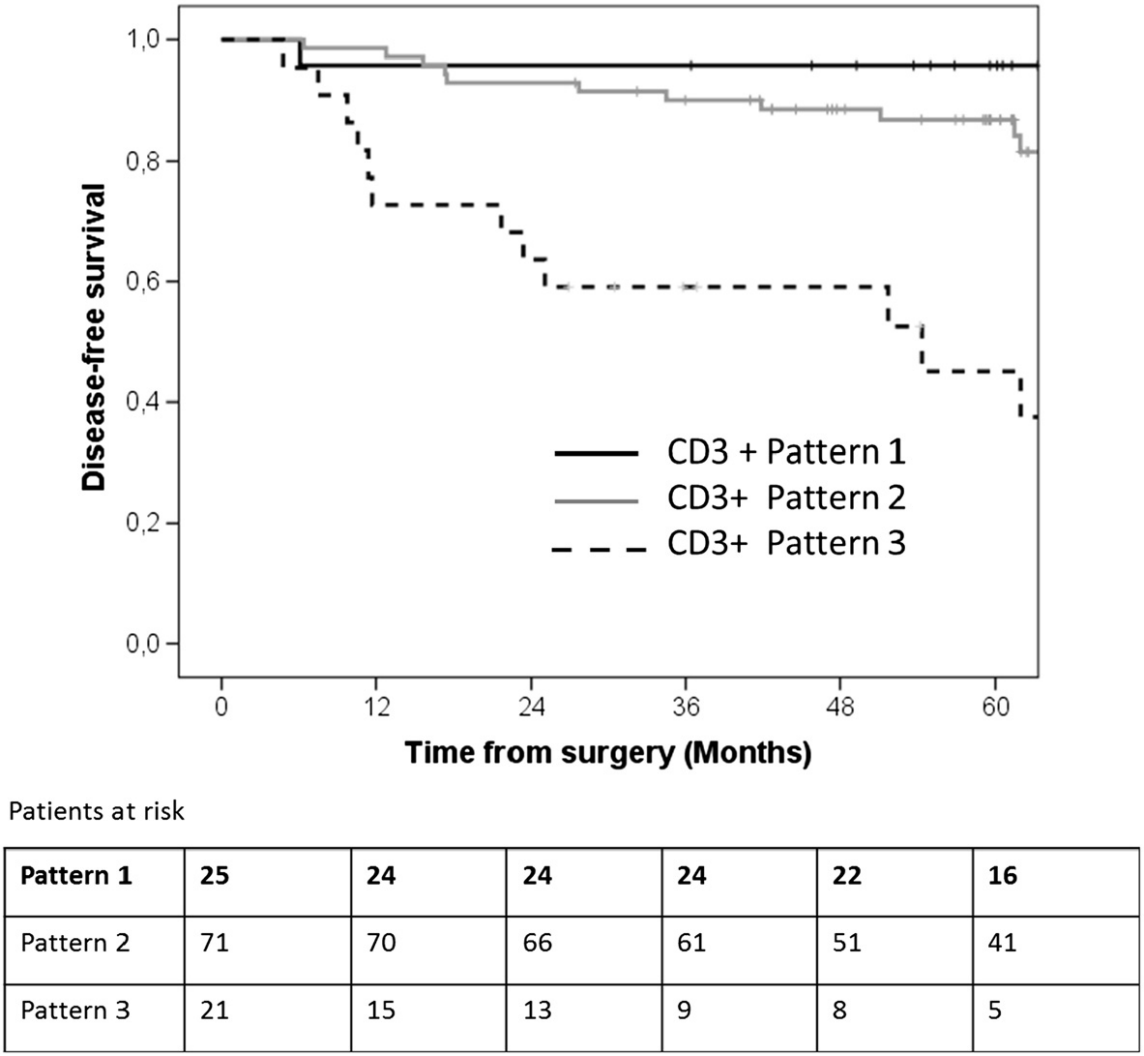
Kaplan Meier disease-free survival curve according to CD3+ LQLI pattern.

On multivariate analysis, LQLI pattern for CD3^+^ cells (HR: 6.02; 95% CI: 2.74-13.18; *P* < 0.001) and metastatic lymph node ratio (HR: 6.14; 95% CI: 2.32-16.2; *P* < 0.001) were independently associated with DFS (Table 4). Patients with high CD3^+^ cell densities on TMA had a better DFS than patients with low CD3^+^ cell densities (92% and 90% versus 80% and 74% at 3 and 5 years; *P* = 0.04; Figure 6). However, this factor was not significant in multivariate analysis. Patients displaying pattern 3 had worse outcomes than those displaying pattern 1 or 2, regardless of the antibody (directed against CD3 or CD45R0) used. With immunostaining for CD3, three- and five-year DFS was 96% and 96%, respectively, in patients with pattern 1, 90% and 86% in patients with pattern 2, and 59% and 45% in patients with pattern 3 (Figure 7).

**Table 4:** Multivariate analysis of disease-free survival

**Variable**	**Multivariate analysis**		
	**HR**	**95% CI**	***P***
Metastatic LNR	6.14	2.32-16.2	<0.001
CD3^+^ LQL pattern	6.02	2.74-13.18	<0.001

*LNR* lymph node ratio, *HR* hazard ratio, *CI* confidence interval.

## Discussion

Many studies have already demonstrated the prognostic value of lymphoid infiltration (LI) in CRC [3-5,11-13]. However, LI evaluation is still not used in daily practice to determine whether a patient should receive adjuvant treatment. Automated counts on virtual immune-stained slides from patients with CRC have already been investigated in other studies [12,13]. We developed an automated method of LI evaluation suitable for use in routine clinical practice.

Our automated method has several specific features accounting for its robustness and high level of reproducibility. The size of area analyzed (more than 14 times the area of a TMA core) limits the impact of count variability due to the heterogeneity of LI. Furthermore, taking into account the demonstration, by Gallon *et al.*, of the prognostic value of lymphocyte density in a region adjacent to the tumor, the method developed here includes both tumor tissue and the surrounding non tumor tissue [13]. Finally, we decided to focus on the variation of lymphocyte density across the invasive front, from the non tumor region to the centre of the tumor, rather than obtaining a mean value for one or two areas. Interestingly, we were able, with a sample of 117 patients with stage II or III CRC, to identify three different curve patterns and to associate these patterns with long-term outcome. One of the original findings of this study was the presence of a peak of lymphocyte density around the invasive front of the tumor in the curves or more than half (61%) the study population. This finding is reminiscent of the high lymphocyte densities found at the invasive margin of colorectal liver metastases, which are also associated with a favorable outcome [20].

Lymphocyte density has been assessed by several methods, including the counting of lymphocytes on H&E-stained sections and immunostaining. It has been evaluated on TMA and on whole slides [5,11-13,21]. Immunostained slides have been evaluated by a pathologist [22] or by dedicated software. A recent study confirmed that the results of automated counting of tumor-infiltrating lymphocytes based on image analysis are closely correlated with the semi-quantitative results obtained by a pathologist [23]. Software can be used to evaluate the percentage of the study area that is immunoreactive [11] or to identify lymphocytes by segmentation [12]. Such segmentation-based analysis may prove difficult if the lymphocytes are located close together [21]. We obtained identical patterns for analyses of the percentage of the area displaying immunoreactivity and for analyses based on the identification of positive cells.

In most studies, receiver operating characteristic (ROC) curve analysis, with survival as the end-point, is used to determine the cutoff values. This method is problematic in that the mean values obtained cannot be used for patients of other series or in routine practice. This drawback does not apply to LQLI patterns, which are not dependent on the definition of a single threshold value. The LQLI method had a better prognostic value than counts based on the use of TMA technology, and the results obtained were independent of the analysis area. The LQLI method was also feasible for all tumor subtypes, provided that the invasive front was included in the paraffin-embedded block. The only processes requiring direct human intervention in the LQLI method are the positioning of the rectangle for analysis on the virtual slides and determination of the curve pattern. Interobserver reproducibility was excellent (kappa = 0.93).

Microsatellite instability has been shown to be predictive of a favorable outcome, even in the absence of adjuvant therapy, in colorectal cancer. Tumors displaying microsatellite instability are known to have rich lymphoid infiltrates [24-26]. It is therefore important to consider microsatellite status when assessing LI and prognosis. In our study, only one of the 10 patients with MSI-positive tumors had a low density of lymphocytes both in and around the tumor (pattern 3). CD3^+^ cell density was higher in the MSI^+^ group than in tumors without MSI, and the other nine patients displayed LI patterns 1 and 2, but these differences were not statistically significant. No relapses were recorded in the MSI-positive group, but MSI status was not identified as significantly predictive of disease-free survival, possibly due to the small number of patients with MSI-positive tumors.

Another important feature of LI is the location of lymphocytes within the tumor. Indeed, the infiltration of cancer cell networks by CD8^+^ T cells has been reported to be of prognostic value in colorectal cancer [27]. Kayser *et al.* have shown that stromal T cells affect survival in patients with non-small cell lung cancer [28]. We did not investigate the prognostic significance of the location of the lymphocytes within the tumor (stroma versus epithelial tumor cells) with our method. Little is currently known about the precise role of lymphocyte location and its impact on prognosis, and further investigations and confirmation in a different subset of solid tumors are required before this method can be adopted as a standard approach.

By contrast, a number of studies have investigated the nature of the lymphocytes infiltrating tumors. Galon *et al.* analyzed four lymphoid markers (CD3, CD8, CD45R0, granzyme B) and found that combined analysis of CD3^+^ and CD45RO^+^ lymphocyte densities in the center of the tumor and the invasive margin had the best prognostic value [13]. In an independent series, FoxP3 was the only prognostic marker identified, with CD45RO and CD8 significant only in univariate analysis [12]. Both these studies were performed on tissue arrays. The innate immune response may also play an important role in tumor control, as shown by the prognostic value of the density of tumor-infiltrating NK cells and macrophages [29]. Moreover, the respective proportions and the location of the various leukocyte subpopulations with respect to the tumor may be important, as already demonstrated for breast carcinomas [30].

## Conclusion

In conclusion, we describe an original method (LQLI) for the evaluation of LI, plotted as a function of the distance from the invasive front of the tumor. This method is reproducible for a given tumor, and has a better prognostic value than CD3^+^ cell counts on TMA, which is highly variable and less reliable at the individual level. It is easy to use, and results for virtual slides can be obtained in less than two minutes. The LQLI method thus appears to be a powerful tool for investigations of the anti-tumor immune response. Further studies are required to determine which leukocyte subpopulation has the best prognostic value in large series of patients.

## Abbreviations

CRC: Colorectal cancer; LQLI: Linear quantification of lymphocyte infiltration; LI: Lymphocyte infiltration; MSI: Microsatellite instability; H&E: Hematoxylin and eosin; TMA: Tissue microarray; FFPE: Formalin-fixed paraffin-embedded; CI: Confidence interval; HR: Hazard ratio.

## Competing interests

The authors declare that they have no competing interests.

## Authors’ contributions

MAA & JFE conceived the experiment, collected and analyzed data, and wrote the manuscript. JBB collected data. MAA and AB were responsible for the statistical analysis, CJ, CP, RM and BN analyzed data. All the authors approved the manuscript.
